# Hypercoagulability in Cushing’s syndrome: past, present, future

**DOI:** 10.20945/2359-4292-2025-0095

**Published:** 2025-11-24

**Authors:** Amit Akirov, Maria Fleseriu

**Affiliations:** 1 Institute of Endocrinology, Beilinson Hospital, Rabin Medical Center, Petah Tikva, Israel; 2 Faculty of Medicine, Tel Aviv University, Tel Aviv, Israel; 3 Pituitary Center, Departments of Medicine and Neurological Surgery, Oregon Health & Science University, Portland, OR, United States

**Keywords:** Cushing’s syndrome, Thrombophilia, Thromboembolism

## Abstract

Cushing’s syndrome is a chronic disorder characterized by prolonged glucocorticoid exposure, leading to significant multisystem complications. Multiple epidemiological studies have demonstrated a substantially elevated risk of venous thromboembolism in patients with Cushing’s syndrome, including deep vein thrombosis and pulmonary embolism, particularly during active disease, the perioperative period, but more importantly also after biochemical remission. Hypercortisolism promotes a hypercoagulable state through multiple mechanisms, including persistent endothelial dysfunction, increased procoagulant factors such as von Willebrand factor and factor VIII, impaired fibrinolysis, and venous stasis. Additionally, common comorbidities in Cushing’s syndrome, such as obesity, hypertension, and diabetes, further amplify thrombotic risk. Given these findings, recent consensus recommends thromboprophylaxis for most patients with Cushing’s syndrome, with anticoagulation therapy initiated at diagnosis, continued perioperatively, and extended post-remission when appropriate in patients both after surgery and also in patients on medical therapy. Low molecular weight heparin is the preferred anticoagulant, while direct oral anticoagulants require further investigation in patients with Cushing’s syndrome. Despite these recommendations, clinical practice varies significantly across centers and countries, highlighting the need for standardized thromboprophylaxis protocols. Future research should focus on refining risk stratification models, optimizing prophylaxis duration, and evaluating the long-term thrombotic risk in Cushing’s syndrome remission. Additionally, studies exploring the safety and efficacy of direct oral anticoagulants and personalized medicine approaches through biomarker-driven strategies may further improve patient outcomes. Addressing these gaps will enhance thromboembolism prevention strategies in Cushing’s syndrome and ultimately may reduce morbidity and mortality in this high-risk population.

## INTRODUCTION

Endogenous Cushing’s syndrome (CS) is classified as adrenocorticotropic hormone (ACTH)-dependent or ACTH-independent. Adrenocorticotropic hormonedependent cases (60% to 70%) are primarily due to Cushing’s disease (CD) from a corticotroph pituitary adenoma (80% to 90%) or ectopic ACTH secretion (6% to 10%) linked to neuroendocrine tumors. Adrenocorticotropic hormone-independent CS (20% to 30%) is mainly caused by adrenal adenomas, carcinomas, or bilateral adrenal hyperplasia (^1^,^2^).

Cushing’s syndrome is a chronic disorder caused by prolonged glucocorticoid exposure, with an estimated incidence of 2 to 8 cases per million people annually and a prevalence of 57 to 79 cases per million, though underdiagnosis is likely. It predominantly affects females, with a female-to-male ratio of approximately 4:1, and is most commonly diagnosed between ages 30 and 49, though cases have been reported from ages 5 to 75 (^1^,^2^).

Excess cortisol affects multiple systems, leading to hyperglycemia, muscle and bone loss, weight gain, hypertension, immune suppression, neurocognitive impairment, osteoporosis, and mood disorders such as depression (^1^). Mortality is higher than in general population and infections and cardiovascular disease are the leading causes of death, while thromboembolic events, including pulmonary embolism (PE) and deep vein thrombosis (DVT), also contribute significantly (^1^,^3^). Common CS-associated conditions, including hypertension, diabetes mellitus, osteoporosis with fractures, immune suppression, and cancer, are known risk factors for venous thromboembolism (VTE) (^4^,^5^). Additionally, surgery and hospitalizations, which are often necessary for CS treatment, further increase VTE risk (^6^). Surveys over the last decades showed that awareness is expanding on risk of VTE in patients with CS, however, albeit increasing, a low percentage of patients has been treated prophylactically, with large centers and countries dissimilarities (^7^). The Pituitary Society Consensus guideline suggested increased perioperative anticoagulants use in patients with CS; however, no consensus was reached regarding the duration of anticoagulation after remission (^8^).

This year, a Delphi position statement on thromboprophylaxis in endogenous CS recommends anticoagulant therapy at diagnosis, during the perioperative period, and for several months post-remission, provided there are no contraindications (^9^).

This review aims to summarize the epidemiology, pathophysiology, management strategies and future directions for VTE prevention in patients with CS.

## EPIDEMIOLOGY

Multiple studies have demonstrated an increased risk of VTE in patients with CS, particularly during active disease, the postoperative period, and even after achieving remission. Interestingly, an increased risk of VTE events is also observed in patients with exogenous CS (^10^).

### Active Cushing’s syndrome

Patients with active CS are at a significantly increased risk for VTE compared to the general population. A Danish population-based study found that the risk of VTE in patients with CS was 2.6 times higher than in matched controls, with the highest risk occurring around the time of diagnosis (^11^). Similarly, a Swedish nationwide study reported a standardized incidence ratio (SIR) of 11.5 for thromboembolism during 3 years before diagnosis, suggesting that hypercoagulability is present even before treatment (^12^). A systematic review and meta-analysis by Wagner and cols. further supported these findings, reporting an odds ratio (OR) of 17.82 for VTE in patients with CS compared to the general population (^13^). Additionally, a nationwide cohort study conducted by our group found that the 5-year risk of VTE in patients with CS was nearly five times higher than in controls from the general population with a hazard ratio (HR) of 4.71 (^14^). The study identified age ≥ 60 years, hypertension, ischemic heart disease, kidney disease, and prior VTE as significant predictors of thromboembolic events (^14^). Collectively, these studies highlight that CS itself, independent of treatment, predisposes patients to a hypercoagulable state, likely due to increased levels of procoagulant factors such as von Willebrand factor (vWF) and factor VIII, as well as impaired fibrinolysis.

When compared to patients with nonfunctioning pituitary adenomas (NFPA), the risk of VTE remains higher in those with CS. A multicenter cohort study from the Netherlands found that patients with ACTHdependent CS had a postoperative VTE incidence of 3.4% within 3 months of surgery, a significantly higher rate than in patients with NFPA, suggesting that the endogenous cortisol excess in CS contributes to a higher thrombotic risk compared to other pituitary disorders, even when controlling for risk related to surgical interventions (^15^).

The risk of VTE in CS has also been compared to that in patients with mild autonomous cortisol secretion (MACS), a condition characterized by subtle cortisol excess without overt CS symptoms in patients with adrenal adenomas. A retrospective analysis of the American College of Surgeons National Surgical Quality Improvement Program (ACS-NSQIP) database found that postoperative VTE was significantly more common in patients with CS than in those undergoing adrenalectomy for MACS (2.6% *versus* 0.9%; p = 0.007). Patients with CS were younger, had higher body mass index, and were more likely to have diabetes mellitus (p < 0.001). They also experienced longer operative times and hospital stays (p < 0.001), which were associated with increased VTE risk (^16^). These findings indicate that mild cortisol excess carries a lower thrombotic risk than overt hypercortisolism, further reinforcing the role of cortisol excess in promoting coagulation abnormalities.

### Perioperative period

The postoperative period represents a particularly high-risk window for VTE in patients with CS. A Danish study reported an extremely high risk of VTE in the 3 months following surgery, with a HR of 59.9 (^11^). Similarly, a Swedish nationwide study found a peak in thromboembolism incidence from diagnosis until 1 year post-remission, with an SIR of 18.3 (^12^). A large, US single-center retrospective study by Suarez and cols. confirmed an increased risk of thromboembolism 30 to 60 days postoperatively (^17^), while Manetti and cols. reported that this risk remained elevated beyond 1 year after pituitary surgery (^18^). Findings from the European Registry on Cushing’s Syndrome (ERCUSYN) further emphasize the importance of postoperative risk, as 87% of the 95 VTE events recorded among 2,174 patients occurred after surgery, with nearly half occurring within 6 months (^19^). Key risk factors here included male sex, high urinary free cortisol levels at diagnosis, and multiple surgeries (^19^). A recent systematic literature review that included 25 relevant studies reported that the pooled incidence of postoperative VTE in patients undergoing transsphenoidal surgery for CD was 2% (58/2,997), while VTE-related mortality was 0.2% (6/2,077). No cases of postoperative VTE were reported in 191 patients undergoing adrenalectomy for benign ACTHindependent CS. Most reported VTE cases were DVT (48%), while cerebral venous sinus thrombosis was rare (6%). Perioperative thromboprophylaxis strategies varied significantly, but studies comparing different anticoagulation approaches suggested that extended prophylaxis was more effective than shorter-duration regimens (^20^). These studies highlight the need for extended thromboprophylaxis in the perioperative period, as patients with CS may remain at elevated risk for months following surgical intervention.

### Disease remission

Even after achieving biochemical remission, patients with CS continue to face an increased risk of thromboembolism. A Swedish study found that while the SIR for VTE declined over time, it remained significantly elevated at 4.9 during long-term remission (^12^). Similarly, Manetti and cols. reported that coagulation abnormalities persist beyond 1 year postoperatively, suggesting that the prothrombotic state in CS may not fully resolve even after successful treatment (^18^). These findings underscore the need for ongoing monitoring and individualized risk assessment in patients with a history of CS, even after remission has been achieved.

Together, these studies highlight that patients with CS carry an elevated risk for VTE not only during active disease, but also risk is significantly increased in the postoperative period and even during long-term remission.

## PATHOPHYSIOLOGY

**Figure 1:**
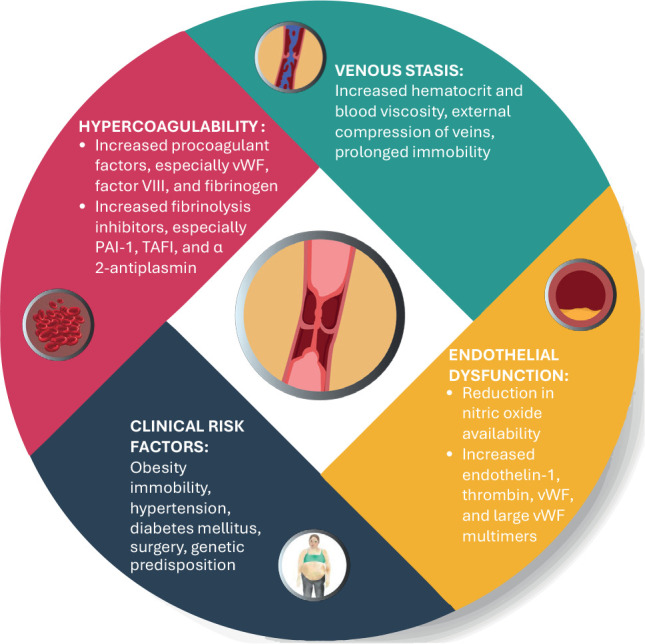
Pathophysiology of venous thromboembolism in patients with Cushing’s syndrome. Hypercortisolism promotes a hypercoagulable state through multiple mechanisms, including endothelial dysfunction, increased procoagulant factors, impaired fibrinolysis, and venous stasis.

PAI-1: plaminogen activator inhibitor-1; TAFI: thrombin-activatable fibrinolysis inhibitor; wWF: von Willebrand factor.

Clinical studies have demonstrated significant abnormalities in coagulation and fibrinolysis in patients with hypercortisolism, contributing to an increased risk of thromboembolic events (^21^). Hypercortisolism promotes a hypercoagulable state through multiple mechanisms, including endothelial dysfunction, increased procoagulant factors coupled with impaired fibrinolysis, and venous stasis (^22^). These changes align with Virchow’s triad, which describes the key contributors to thrombosis: vascular abnormalities, hypercoagulability, and stasis (^21^) (**Figure 1**).

### Endothelial dysfunction

Factors such as obesity, diabetes mellitus, hypertension, dyslipidemia, and insulin resistance, all frequently observed in CS, contribute to impaired endothelial function by disrupting nitric oxide availability and increasing endothelin-1 levels (^1^,^23^-^25^). Elevated markers of endothelial dysfunction, such as intercellular adhesion molecule-1 and serum N-acetylβ-glucosaminidase activity, have been reported in patients with hypercortisolism (^24^). Additionally, increased levels of vWF and unusually large vWF multimers indicate endothelial activation, further enhancing the thrombotic potential (^26^,^27^).

### Hypercoagulability and imbalance in coagulation factors

Cushing’s syndrome is characterized by increased plasma levels of procoagulant factors, including factors II, V, VIII, and IX, alongside shortened partial thromboplastin time (PTT), likely due to elevated factor VIII levels (^15^,^18^,^28^,^29^). The persistence of elevated vWF, factor VIII, and factor IX even after remission suggests a lasting prothrombotic state (^29^). While some studies have reported increased levels of anticoagulants such as protein C, protein S, and antithrombin, this appears to be a compensatory response to heightened coagulation activity (^28^,^30^). However, hypercortisolism also impairs fibrinolysis by increasing inhibitors such as plasminogen activator inhibitor-1 (PAI-1) and thrombin-activatable fibrinolysis inhibitor (TAFI), further promoting clot formation (^28^,^29^,^31^,^32^). Studies have shown increased clot formation speed and clot strength in patients with active CS, particularly in those with obesity, reinforcing the role of cortisol excess in accelerating thrombogenesis (^33^). Inflammatory and prothrombotic endothelial damage has also been observed in patients with CS even after remission: the early remission phase following the correction of hypercortisolism is marked by persistent low-grade inflammation, lasting up to 1 year post-surgery, and patients with CS who also have obesity or hyperglycemia are at a higher risk of elevated inflammatory markers during the postoperative period, potentially increasing their susceptibility to thromboembolism during this time (^34^).

### Venous stasis

Venous thrombi commonly form in areas of slow blood flow, such as the deep veins of the legs (^35^). Patients with CS may exhibit increased hematocrit and blood viscosity, which can lead to reduced venous flow and promote clot formation (^35^,^36^). Additionally, external compression of veins and prolonged immobility, common in severely affected CS patients, further increase the risk of stasis-related thrombotic events (^21^).

### Comorbidities and clinical risk factors

Thromboembolic events in CS are often triggered by additional risk factors, including surgery, malignancy, as well as conditions such as arterial hypertension, diabetes mellitus, obesity, and smoking that contribute to a prothrombotic state. Studies have found that many patients with VTE unrelated to surgery had multiple acquired risk factors, such as metabolic disorders and infections (^37^). In addition, genetic predispositions, such as polymorphisms in the VWF gene promoter and inherited thrombophilic defects like factor V Leiden and prothrombin gene mutations, can exacerbate the hypercoagulable state in CS (^38^). Moreover, patients with ectopic CS and adrenal carcinoma face a heightened risk of VTE due to underlying malignancy (^21^). The combination of high vWF levels, genetic predisposition, and acquired risk factors likely amplifies the risk of thrombosis in CS.

## THROMBOPROPHYLAXIS MANAGEMENT

**Figure 2:**
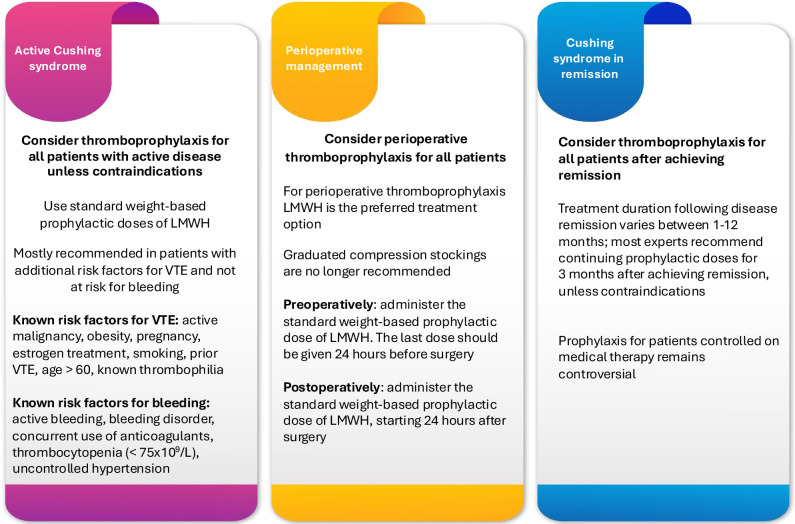
Thromboprophylaxis in patients with Cushing syndrome.

Current clinical practices for thromboprophylaxis management in patients with CS vary between countries and even centers (^8^,^22^,^39^); a study across EU Reference Network Rare Endocrine Conditions (Endo-ERN) reference centers reported notable differences in treatment protocols (^40^). The recent Delphi consensus on VTE management in CS which included endocrinologists, epidemiologists, neurosurgeons and hematologists highlights the critical need for thromboprophylaxis in most patients due to their elevated thrombotic risk. The recommendations outlined in the position statement aim to standardize care and enhance patient outcomes (^9^) (**Figure 2**).

### Active Cushing’s syndrome

According to aforementioned position statement, thromboprophylaxis should be considered for all patients with CS but should only be initiated after confirming the diagnosis, except in cases of severe hypercortisolemia. The decision to administer anticoagulation must be carefully weighed in high-risk populations, as certain conditions significantly increase the risk of bleeding, including active bleeding, acquired bleeding disorders (e.g., liver failure), concurrent use of anticoagulants, acute stroke, thrombocytopenia (platelet count <75 × 10^9^/L), uncontrolled hypertension (blood pressure >200 mmHg systolic or > 120 mmHg diastolic), untreated inherited bleeding disorders (e.g., hemophilia, von Willebrand disease), or recent or planned surgical procedures. On the other hand, thromboprophylaxis should be strongly considered in patients with additional risk factors, such as active malignancy, sedentary lifestyle, smoking, estrogen therapy, pregnancy, age over 60, known thrombophilia, obesity, significant comorbidities (e.g., cardiovascular disease, pulmonary disorders, acute infections, inflammatory conditions), or a personal or family history of VTE. While the use of oral estrogen is not contra-indicated in patients with active CS, temporarily discontinuing oral estrogen therapy in female patients prior to surgery should be considered and alternate modes of estrogen deliveries or other contraceptive measures without oral estrogen initiated as needed (^41^). Hospitalized patients with active CS should receive thromboprophylaxis, regardless of the reason for admission or the severity of hypercortisolism, as long as no contraindications exist.

Low molecular weight heparin (LMWH) in standard weight-based prophylactic doses is the preferred anticoagulant for thromboprophylaxis in patients with CS, similar to the American Society of Hematology guidelines on the management of VTE in patients with cancer (^42^). Direct oral anticoagulants (DOACs) are not currently approved for this indication and should be used with caution. In contrast with previous guidelines, graduated compression stockings are not recommended at this time, as a recent systematic review found no additional benefit in preventing VTE or reducing mortality and their use poses a risk of skin complications, which is particularly relevant in patients with CS with fragile skin (^43^).

### Inferior petrosal sinus sampling

Although some reports have indicated a higher incidence of VTE events with inferior petrosal sinus sampling (IPSS), the decision to administer periprocedural anticoagulation for all patients remains controversial (^44^,^45^). In the latest Delphi position statement, consensus was reached that patients not already receiving anticoagulation should be started on it approximately 12 hours before IPSS. For those already on anticoagulation, prophylactic doses of LMWH can be maintained during the procedure, but higher doses should be withheld 24 hours before and resumed 48 hours after the procedure. Direct oral anticoagulants regimens vary, with discontinuation 24 to 72 hours before the procedure, bridging with LMWH, and resumption 48 hours post-procedure.

### Perioperative management

If thromboprophylaxis was not started at the time of CS diagnosis, its perioperative use should be re-evaluated for all patients with CS undergoing surgery, especially in more severe cases, provided there are no clear contraindications ^9^. Preoperatively, the standard weight-based prophylactic dose of LMWH should be administered, with the last dose given 24 hours before surgery. Postoperatively, thromboprophylaxis should resume 24 hours after surgery and LMWH remains the preferred anticoagulant in this setting.

Aspirin has been also previously suggested perioperatively to prevent VTE, but experience in patients with CS is scarce (^46^,^47^).

### Disease remission

For patients who have achieved biochemical remission, thromboprophylaxis should generally continue for 3 months unless contraindications exist. However, expert opinions varies for a treatment duration ranging from 1 to 12 months post-remission. In cases in which the patient has no additional thrombotic risk factors, thromboprophylaxis may not be necessary after biochemical remission, but there is a lack of studies on this topic. In patients with CS who have achieved remission, oral estrogen use immediately post-remission should be carefully evaluated due to the potential ongoing increased risk of thromboembolism, though further research is needed to gain a clearer understanding of this concern. Alternative therapies or other deliveries (e.g., transdermal) to oral estrogen should be explored to help mitigate this risk.

It remains unclear whether medical management of CS influences coagulation risk beyond achieving biochemical remission. Short-term biochemical remission with a combination of pasireotide, cabergoline, and ketoconazole did not show significant improvement in hypercoagulable markers (^29^). However, emerging data suggest some direct effects on coagulation factors. While subcutaneous pasireotide treatment for 6 to 12 months did not significantly alter clotting factors (^48^), relacorilant, a glucocorticoid receptor selective blocker in development, has been shown after 3 to 4 months to induce significant mean changes in factor VIII (-18.9%; p = 0.022), aPTT (+1.5 s; p = 0.046), and platelet count (-68.8 × 10^9^/L; p < 0.0001) (^49^). Interestingly, vWF remained largely unchanged. Differences in the effects of these drugs on coagulation factors may be attributed to their distinct mechanisms of action, with pasireotide carrying an additional risk of hyperglycemia.

If VTE is diagnosed in a patient with CS, therapeutic anticoagulation should be administered for 3 to 6 months as per guidelines for VTE treatment, followed by prophylactic dosing for at least three months after hypercortisolism is resolved. However, treatment decisions should be individualized based on ongoing risk factors.

### Risk of bleeding

While the primary haemostatic concern in CS is hypercoagulability and the associated risk of VTE, there is also a notable risk of bleeding. This risk is particularly evident in the context of gastrointestinal complications, such as peptic ulcers and gastrointestinal bleeding, which are associated with hypercortisolism (^11^,^50^), and may also arise following surgical interventions.

Few studies have examined the risk of bleeding in patients with CS who are anticoagulated, either shortor long-term. In patients with brain tumors who underwent various neurosurgical procedures, a metaanalysis on thromboprophylaxis found a slightly increased risk of minor hemorrhagic complications (risk ratio = 2.02), though no significant increase in major bleeding events (^51^).

## FUTURE DIRECTIONS

Future research should focus on refining thromboprophylaxis strategies for patients with CS to optimize patient outcomes while minimizing risks. A major priority is the development of standardized protocols, as current practices vary significantly across centers. Prospective studies are needed to establish the optimal timing, duration, and choice of anticoagulant therapy, particularly given the lack of consensus on thromboprophylaxis duration, which currently ranges from 1 to 12 months post-surgery. Need for anticoagulation for patients in biochemical remission also needs to be further studied. Additionally, defining the period during which patients remain at an elevated thrombotic risk after biochemical remission is crucial to improving long-term management.

Risk stratification algorithms incorporating both general and CS-specific factors, such as the Padua Score or CS-VTE score, could enhance individualized thromboprophylaxis. Implementing these tools may allow clinicians to better assess thrombotic risk and tailor prophylaxis accordingly, reducing unnecessary anticoagulation in low-risk patients while ensuring adequate protection for those at higher risk. Further research is also needed to clarify the role of long-term anticoagulation, as hypercoagulability in CS may persist for months following surgical remission. Understanding the balance between thrombotic and bleeding risks will help guide decisions on extended anticoagulation therapy.

The potential use of DOACs in CS represents another important area for investigation. While DOACs offer advantages in other hypercoagulable states, their use in CS remains limited, and safety data are lacking. A particular concern is the reported higher bleeding risk associated with DOACs compared to LMWH (^52^). Dedicated clinical trials are necessary to evaluate their efficacy, safety, and suitability for CS patients.

Currently, there is insufficient evidence to provide clear recommendations regarding thromboprophylaxis in patients with mild CS or MACS. Further research is needed to determine the thrombotic risk in these populations and guide management strategies accordingly.

Finally, future research should explore personalized medicine approaches through genetic and biomarker-driven strategies to refine thromboprophylaxis. Identifying molecular markers that predict VTE risk could enable a more individualized approach, ensuring that high-risk patients receive appropriate prophylaxis while avoiding unnecessary anticoagulation in those at lower risk. By addressing these gaps, future studies have the potential to improve patient safety, standardize care, and enhance outcomes in this high-risk population.

## Data Availability

datasets related to this article will be available upon request to the corresponding author.
